# West Nile virus illness in Ontario, Canada: 2017

**DOI:** 10.14745/ccdr.v45i01a04

**Published:** 2019-01-03

**Authors:** S Wijayasri, MP Nelder, CB Russell, KO Johnson, S Johnson, T Badiani, D Sider

**Affiliations:** 1 Enteric, Zoonotic and Vector-borne Diseases, Communicable Diseases and Emergency Preparedness and Response, Public Health Ontario, Toronto, ON; 2 Knowledge Services, Public Health Ontario, Toronto, ON; 3 Department of Clinical Epidemiology and Biostatistics, McMaster University, Hamilton, ON

**Keywords:** *Culex*, mosquito, surveillance, epidemiology, public health, climate, West Nile virus

## Abstract

**Background:**

In Canada, the annual incidence rates of West Nile virus (WNV) illness have fluctuated over the last 15 years. Ontario is one of the provinces in Canada most affected by WNV and, as a result, has implemented robust mosquito and human surveillance programs.

**Objective:**

To summarize and discuss the epidemiology of WNV illness in Ontario, Canada in 2017, with comparisons to previous years.

**Methods:**

Case data were obtained from the provincial integrated Public Health Information System. Provincial and public health unit (PHU)-specific incidence rates by year were calculated using population data extracted from intelliHEALTH Ontario.

**Results:**

In 2017, the incidence of WNV illness in Ontario was 1.1 cases per 100,000 population, with 158 confirmed and probable cases reported by 27 of the province’s 36 PHUs. This is the highest rate since 2013, but less than the rate in 2012 (2.0 cases per 100,000 population). Incidence rates in 2017 were highest in Windsor-Essex County and in PHUs in eastern Ontario. While the seasonality is consistent with previous years, the number of cases reported between July and September 2017 was above expected. Most cases were in older age groups (median: 58 years old) and males (59.5% of provincial total); cases with severe outcomes (neurological complications, hospitalizations, deaths) were also disproportionately in older males.

**Conclusion:**

WNV illness continues to be an ongoing burden in Ontario. The increase in the number of cases reported in 2017, and the increased number of PHUs reporting cases, suggests changing and expanding risk levels in Ontario. Continued mosquito and human surveillance, increased awareness of preventive measures, and early recognition and treatment are needed to mitigate the impact of WNV infections.

## Introduction

West Nile virus (WNV) is a mosquito-borne pathogen of public health concern in Canada. The virus was first identified in North America in 1999, with the first human case of WNV illness in Canada confirmed in Ontario in 2002 (1,2). The virus has since become endemic to Canada, with the annual number of cases reported nationally fluctuating over the last 15 years (reaching a high of 2,215 cases in 2007 and a low of five cases in 2010) (3). Cases have been reported in all ten provinces since 2002, with the majority occurring in the prairie and central provinces (4). Ontario (which represents approximately 38.7% of the Canadian population) has reported cases of WNV illness every year since 2002, with epidemics reported in 2002 and 2012 (2,5,6).

In Ontario, *Culex* mosquitoes are primarily responsible for WNV transmission to humans (7). Mosquito development, and the rate of virus replication inside the mosquito, is heavily driven by temperature and geography – they are most active in warmer temperatures and urban environments where catch basins with standing water are widespread (6,7). In 2016, the majority of WNV-positive mosquito pools in Ontario were reported in the Golden Horseshoe and urban areas of southwestern and southeastern Ontario (7). Studies have identified a strong relationship between the number of WNV-positive mosquito pools and the number of confirmed human cases reported each year, highlighting the usefulness of mosquito surveillance in early detection and risk assessment (6,8).

While the majority of cases are asymptomatic, or do not seek medical attention due to mild symptoms, a fraction of those infected develop severe outcomes, including neuroinvasive disease (9,10). Neuroinvasive disease that can present as meningitis, encephalitis or acute flaccid paralysis are difficult to treat and are associated with high morbidity, mortality and long-term sequelae (9,10). Considering that WNV infection can lead to severe illness, and that treatment is only supportive, public health efforts have focused on early detection through mosquito and human surveillance, promotion of preventive measures and increasing awareness (9). An understanding of WNV epidemiology is therefore necessary to inform such public health efforts.

The objective of this report is to summarize and discuss the epidemiology of WNV illness in Ontario, Canada in 2017, with comparisons to previous years.

## Methods

### Population and surveillance case definitions

During the 2017 surveillance period, there were 36 public health units (PHUs) in Ontario that provided local health services within their jurisdictions (11). Under the *Health Protection and Promotion Act*, all PHUs are responsible for case management and reporting of diseases of public health significance in Ontario (12). PHUs classify and report confirmed and probable WNV illness cases using the provincial surveillance case definitions and disease classifications (13).

### Data source

PHUs report WNV illness cases to the province using the web-based integrated Public Health Information System (iPHIS). PHU reports include information on case demographics, exposures, symptoms, hospitalizations and deaths. Details for confirmed and probable cases of WNV illness with episode dates from 2005 to 2017 were extracted from iPHIS. Episode date is an approximation of illness onset, based on the first available date in the following hierarchy: symptom onset, specimen collection, laboratory result or report date.

### Analyses

Descriptive analyses were conducted using SAS 9.3 and Microsoft Excel 2010. Case-level data from iPHIS were used to describe the geographic trends, seasonality, age and sex distributions and clinical outcomes of WNV illness cases in Ontario reported in 2017. To eliminate the skewing effect of the WNV epidemic in 2012, the epidemic year was excluded from historical averages, and four-year historical averages (2013–2016) were used as comparators to 2017 (6). Provincial incidence rates (2005–2016) and PHU-specific incidence rates (2017) were calculated per 100,000 population per year using provincial and PHU population estimates (2005–2016) and projections (2017) obtained from Statistics Canada via intelliHEALTH Ontario. Given the uncertainties with assigning exposure locations, cases reporting travel outside the province during the incubation period were not excluded from the analyses. ESRI ArcGIS^TM^ v10.3 (ESRI, Redlands, California, United States) was used to map WNV illness incidence rates by PHUs for 2017. Rates were grouped into classes using manual classification methods.

### Ethics statement

This manuscript reports on routine surveillance activities and not research, and therefore research ethics committee approval was not required. Data are available upon request via Public Health Ontario at http://www.publichealthontario.ca/en/About/Pages/privacy.aspx.

## Results

### Overall

**Figure 1:**
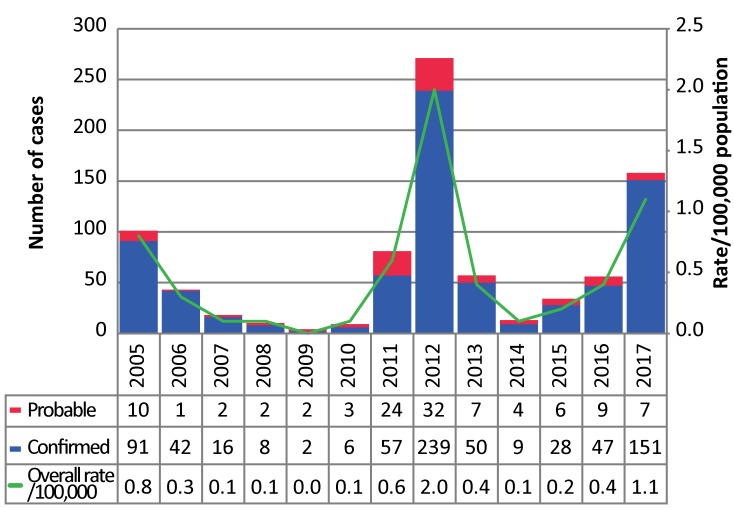
Number of confirmed and probable West Nile virus illness cases and incidence (per 100,000 population), by year, in Ontario, Canada, 2005–2017

In 2017, 158 confirmed and probable WNV illness cases were reported in Ontario, well above the four-year historical average of 40 cases per year. This is the second highest number of cases reported in a single year since 2005, with the number of cases increasing annually since 2014 (**Figure 1**). The incidence rate of WNV illness in Ontario in 2017 was 1.1 cases per 100,000 population, an almost three-fold increase from 2016 (0.4 cases per 100,000 population), but below the incidence rate in 2012 (2.0 cases per 100,000 population).

### Geographic distribution

Twenty-seven PHUs reported WNV illness cases in 2017. This is higher than in the previous four years (2013–2016), during which 13 to 15 PHUs reported cases per year. Of the total cases reported in Ontario in 2017, the majority of cases were reported by Toronto (28/158 cases, 17.7%), followed by Ottawa (20/158 cases, 12.7%) and Windsor-Essex County (20/158 cases, 12.7%). Increases in Ottawa (7.5 times its four-year historical average) and Windsor-Essex County (5.7 times its four-year historical average) were particularly notable (**Table 1**).

**Table 1:** Number of confirmed and probable West Nile virus illness cases reported in 2017, compared to four-year historical averages (2013–2016), by public health unit^a^, Ontario, Canada

Health Unit	2017		2013–2016
	n	%^b^	Four-year average
Toronto	28	17.7	12.5
City of Ottawa	20	12.7	2.7
Windsor-Essex County	20	12.7	3.5
York Region	12	7.6	1.5
Peel Region	10	6.3	4.3
Niagara Region	8	5.1	5.7
Simcoe Muskoka District	7	4.4	1.3
City of Hamilton	6	3.8	3.7
Halton Region	6	3.8	1.5
Leeds, Grenville and Lanark District	6	3.8	0.0
Eastern Ontario	5	3.2	1.0
Grey Bruce	4	2.5	0.0
Durham Region	3	1.9	0.0
Haliburton, Kawartha, Pine Ridge	3	1.9	0.0
Peterborough County-City	3	1.9	0.0
Waterloo Region	3	1.9	0.0
Kingston, Frontenac, Lennox & Addington	2	1.3	0.0
Sudbury and District	2	1.3	1.0
Wellington-Dufferin-Guelph	2	1.3	1.0
Haldimand-Norfolk	1	0.6	0.0
Hastings & Prince Edward Counties	1	0.6	0.0
Lambton County	1	0.6	1.0
Middlesex-London	1	0.6	2.0
Oxford County	1	0.6	1.0
Perth District	1	0.6	0.0
Renfrew County and District	1	0.6	1.0
Timiskaming	1	0.6	0.0
**Total (Ontario)**	158	100.0	40.0

Abbreviation: n, number

^a^ Public health unit refers to the individual’s health unit of residence at the time of illness onset and not necessarily the location of exposure. Location of disease acquisition cannot be attributed to public health unit. Only public health units that reported cases in 2017 are included in this table (n=27)

^b^ Percent (%) is the proportion of total cases reported in Ontario in 2017 (n=158)

**Figure 2:**
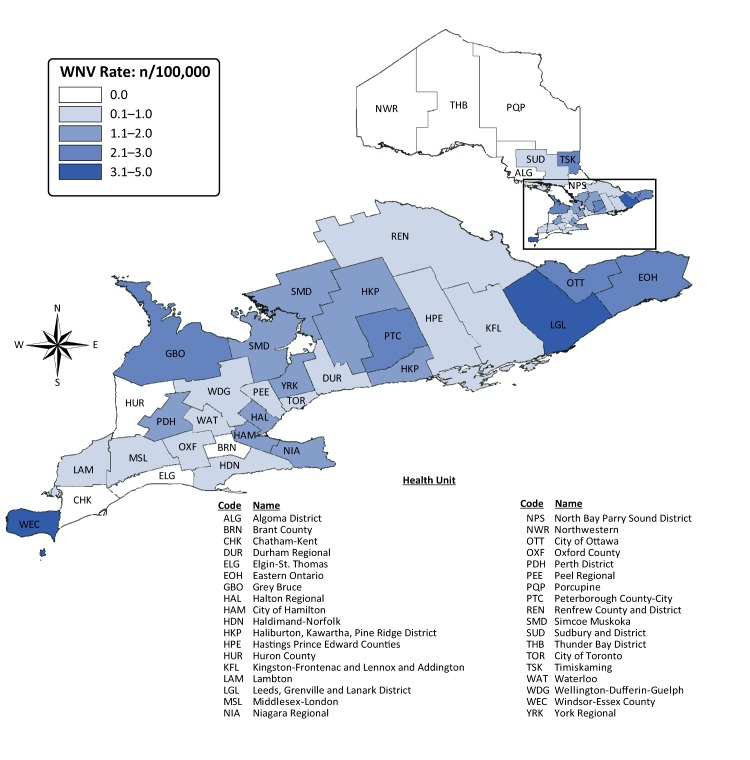
Incidence of West Nile virus illness (per 100,000 population) in 2017, by public health unit^a^, Ontario, Canada Abbreviations: n, number; WNV, West Nile virus ^a^ Public health unit refers to the individual’s health unit of residence at the time of illness onset and not necessarily the location of exposure. Location of disease acquisition cannot be attributed to public health unit

Windsor-Essex County also had the highest incidence rate (4.9 cases per 100,000 population) in Ontario in 2017. High incidence rates were also observed in several low population health units in the eastern region of Ontario, including Leeds, Grenville and Lanark District (3.5 cases per 100,000 population), Timiskaming (3.0 cases per 100,000 population) and Eastern Ontario (2.4 cases per 100,000 population) (**Figure 2**). Of cases reporting an exposure in 2017, 9.2% (13/141) reported travel outside the province during the incubation period.

### Seasonality

**Figure 3:**
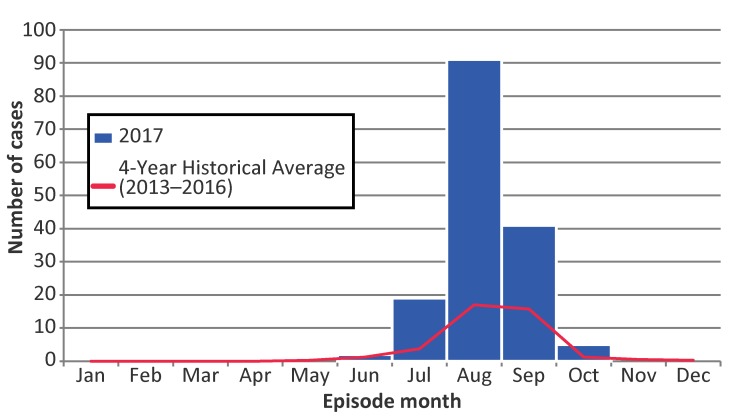
Number of confirmed and probable West Nile virus illness cases reported in 2017, compared to four-year historical averages (2013–2016), by episode month, Ontario, Canada

The majority of cases occurred between July and September 2017, with the highest proportion of cases reported in August (57.6%) (**Figure 3**). The seasonal distribution of cases in 2017 was similar to previous years, peaking in August; however, monthly case counts were more than four times the average of the previous four years for July (observed 19 cases, expected four) and August (observed 91 cases, expected 17).

### Age and sex distribution

**Figure 4:**
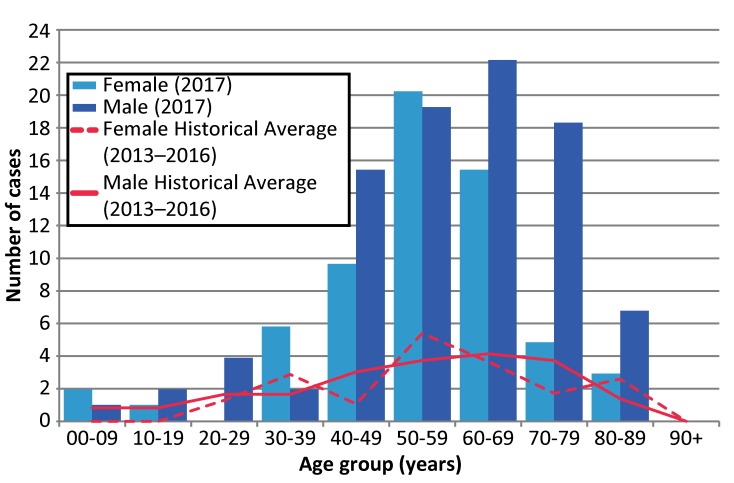
Number of confirmed and probable West Nile virus illness cases reported in 2017 compared to four-year historical averages (2013–2016), by age group^a^ and sex, Ontario, Canada ^a^ Age group refers to the age group (in years) of the individual at the time of illness

Cases of WNV illness in 2017 ranged from five to 89 years old, with most cases in older age groups (median: 58 years old) and males (59.5% of provincial total) (**Figure 4**). In particular, 50.6% of the cases reported in 2017 were 50–69 years old and 51.3% were males over 45 years old. Overall, the age distribution in 2017 follows patterns observed in the previous four years. However, the male to female ratio was higher in the older age groups, particularly in the 40–49 (1.6 times), 60–69 (1.4 times), 70–79 (3.8 times) and the 80–89 (2.3 times) year age groups.

### Clinical outcomes

Of the 158 cases reported in 2017, 38.6% (61 cases) presented with neurological complications, 37.3% (59 cases) presented with non-neurological syndrome, and 2.5% (four cases) were asymptomatic; illness presentation was not specified for 21.5% (34 cases) (**Table 2**). Hospitalization was indicated for 38.6% of cases reported in 2017 (61/158), of which 72.1% (44/61) presented with neurological complications and 14.8% (9/61) presented with non-neurological syndrome; illness was not specified for the remaining 13.1% (8/61). The median age of hospitalized cases was 65 years old (range: 5–80 years old), and 68.9% (42/61) were male. Of the 158 WNV illness cases reported in 2017, nine died (case fatality rate: 5.7%), with WNV illness reported as the underlying or contributing cause for six cases (66.7%). The median age of the nine fatal cases was 79 years old (range: 54–89 years old) and six (66.7%) were male. In comparison, the number of deaths reported between 2013 and 2016 ranged from zero to six per year.

**Table 2:** Number and proportion of confirmed and probable West Nile virus illness cases, by severity of illness and year, in Ontario, Canada, 2013–2017

Severity of Illness	2013		2014		2015		2016		2017	
	n	%	n	%	n	%	n	%	n	%
Clinical Illness (all cases)	57	100	13	100	34	100	56	100	158	100
Asymptomatic	3	5.3	1	7.7	1	2.9	4	7.1	4	2.5
Non-neurological syndrome	22	38.6	3	23.1	12	35.3	13	23.2	59	37.3
Neurological complications	17	29.8	6	46.2	14	41.2	32	57.1	61	38.6
Unspecified illness	15	26.3	3	23.1	7	20.6	7	12.5	34	21.5
Hospitalization^a^	19	33.3	4	30.8	13	38.2	27	48.2	61	38.6
Death^b^	2	3.5	0	0.0	0	0.0	6	10.7	9	5.7

Abbreviation: n, number

^a^ Percent (%) refers to the proportion of total annual cases that were reported as hospitalized due to their disease at the time of data extraction; underreporting of hospitalizations may occur in the integrated Public Health Information System (iPHIS), particularly if the case was hospitalized after follow-up by the public health unit, or if the case was hospitalized for other reasons when they acquired West Nile virus

^b^ Percent (%) refers to the proportion of total annual cases for which a death was reported. Deaths were counted if the case was reported as having died due to their disease at the time of data extraction; variations in follow-up may exist among public health units in determining outcomes for all reportable diseases as well as how the deaths are entered in the type/cause of death fields in iPHIS

## Discussion

The number of WNV illness cases reported in Ontario in 2017 was higher than the previous four years. This trend corresponds to trends in WNV-positive mosquito pools identified in Ontario (14). The seasonality of WNV illness in 2017 was also typical of patterns observed in previous seasons in Ontario and the United States (2,15). However, while the distribution of WNV illness in urban areas of Ontario (Toronto, Ottawa, Windsor-Essex County, York Region and Peel Region) is consistent with mosquito surveillance previously conducted in the province, increases in WNV illness cases were also observed in low population, rural PHUs in eastern Ontario (6,16). The age and sex distribution of cases reported in 2017 were also similar to previous years, with older age groups and males disproportionately affected. The majority of cases with severe clinical outcomes (neurological complications, hospitalizations and deaths) were also older and mostly male, consistent with previous findings that increasing age and being male are risk factors for severe WNV illness outcomes and long-term sequelae (15,17).

### Implications and next steps

While the cause of the increase is not immediately clear, there may some contributing factors. Ontario experienced a relatively warmer (above historical average) 2016–2017 winter, followed by average spring and early summer temperatures (18). The warmer winter temperatures allowed for increased survival of overwintering *Culex* mosquitoes and an increased number of WNV-positive mosquitoes in the spring and summer to start the transmission cycle (19,20). This is consistent with the observation that, while the increase in 2017 was above expected, average temperatures in spring and early summer were not high enough to allow for quicker mosquito development and virus amplification to reach levels similar to 2012 (20,21).

In terms of next steps, there are several public health implications. The 2017 surveillance period highlights the important role of robust and comprehensive surveillance data in WNV prevention and control efforts. Given that temperature is a driving factor in mosquito development and virus amplification, monitoring temperature in conjunction with ongoing mosquito and human surveillance is necessary for early detection and to assess the fluctuating risks of WNV transmission. Mosquito surveillance conducted over several years, particularly in the eastern PHUs, is needed to determine if risk levels are changing in this region and in the province. Such surveillance data are essential to informing targeted public health actions, such as increasing awareness and education related to preventive measures and early recognition, particularly in the older portions of the population and those at risk of severe disease.

### Limitations

As with most passive surveillance systems, the true incidence of disease is underreported due to a variety of factors, such as disease awareness, health care seeking behaviours and variations in clinical testing. Therefore, the incidence of WNV illness is likely underestimated, and skewed toward cases with severe clinical symptoms and outcomes. Given that the majority of WNV cases are asymptomatic or have mild symptoms, and are likely not captured by surveillance, estimating the true burden of WNV infections in the province is particularly challenging. The Public Health Agency of Canada has estimated that 18,000–27,000 WNV infections in Canada may have gone unreported or were unrecognized between 2002 and 2013 (4). As well, the geographic distributions presented in this report are based on the PHU of residence of the case at the time of illness onset and are not necessarily the location of exposure. Exposure locations reported in iPHIS (including travel-related exposure) are not sufficient to determine location of acquisition.

### Conclusion

The number of WNV illness cases reported in Ontario has risen in recent years. While variations in vector biology, weather and human activity make predicting the extent and impact of WNV challenging, it is expected that as ambient temperature increases, the number of WNV illness cases in Ontario and Canada may increase. Continued and strengthened mosquito and human surveillance, public health action to increase awareness of preventive measures, and clinical care focused on early recognition and treatment, will all help to mitigate the impact of WNV illness in Canada.
